# The Absence of MIST1 Leads to Increased Ethanol Sensitivity and Decreased Activity of the Unfolded Protein Response in Mouse Pancreatic Acinar Cells

**DOI:** 10.1371/journal.pone.0028863

**Published:** 2011-12-28

**Authors:** Sruthi Alahari, Rashid Mehmood, Charis L. Johnson, Christopher L. Pin

**Affiliations:** 1 Children's Health Research Institute, London, Ontario, Canada; 2 Departments of Paediatrics and Physiology and Pharmacology, London Health Sciences Centre, University of Western Ontario, London, Ontario, Canada; Texas A&M University, United States of America

## Abstract

**Background:**

Alcohol abuse is a leading cause of pancreatitis in humans. However, rodent models suggest that alcohol only sensitizes the pancreas to subsequent insult, indicating that additional factors play a role in alcohol-induced pancreatic injury. The goal of this study was to determine if an absence of MIST1, a transcription factor required for complete differentiation of pancreatic acinar cells in mice, increased the sensitivity to alcohol.

**Methods:**

Two to four month-old mice lacking MIST1 (*Mist1^−/−^*) or congenic C57 Bl6 mice were placed on a Lieber-DeCarli diet (36% of total kcal from ethanol and fat), a control liquid diet (36% kcal from fat) or a regular breeding chow diet (22% kcal from fat). After six weeks, pancreatic morphology was assessed. Biochemical and immunofluorescent analysis was used to assess mediators of the unfolded protein response (UPR).

**Results:**

Ethanol-fed *Mist1^−/−^* mice developed periductal accumulations of inflammatory cells that did not appear in wild type or control-fed *Mist1^−/−^* mice. Wild type mice fed diets high in ethanol or fat showed enhancement of the UPR based on increased accumulation of peIF2α and spliced XBP1. These increases were not observed in *Mist1^−/−^* pancreatic tissue, which had elevated levels of UPR activity prior to diet exposure. Indeed, exposure to ethanol resulted in a reduction of UPR activity in *Mist1^−/−^* mice.

**Conclusions:**

Our findings suggest that an absence of MIST1 increases the sensitivity to ethanol that correlated with decreased activity of the UPR. Therefore, events that affect the expression and/or function of MIST1 may be confounding factors in pancreatitis.

## Introduction

Chronic alcohol abuse is a leading cause of health issues in North America, increasing the risk of liver disease, hypertension, and cancer. Excessive alcohol consumption accounts for approximately 40% of all cases of chronic and acute pancreatitis, a debilitating disease that affects more than 100,000 people in North America [1], [2]. While a large proportion of acute pancreatitis cases are associated with alcohol abuse, only a small percent of heavy alcohol abusers develop pancreatitis [2] and ethanol administration alone does not initiate pancreatitis in rodent models [3], [4], [5]. Therefore, it is believed that ethanol sensitizes the pancreas to injury. Alternatively, ethanol can exacerbate the effects of other contributors to pancreatic injury, such as a genetic predisposition. A number of studies have identified altered acinar cell physiology in response to ethanol feeding including increased NFκB signaling, altered Ca^2+^ handling and redistribution of proteins involved in SNARE-mediated exocytosis [5], [6]. Recently, the importance of X-box binding protein 1 (XBP1) was examined in the context of ethanol-induced sensitivity to pancreatitis [7]. XBP1 is an important mediator of the inositol-requiring enzyme 1 (IRE1) signaling pathway, one of three such pathways that constitute the unfolded protein response and include PKR-like ER kinase (PERK) and activating transcription factor 6 (ATF6) (reviewed in [8]). When the UPR is triggered by altered Ca^2+^ concentrations or a buildup of unfolded protein in the ER, IRE1 is activated and acts as an endonuclease for *Xbp1* mRNA [9], [10]. Chronic ethanol feeding of wild type (WT) mice led to up-regulation of XBP1, and mice heterozygous for *Xbp1* (*Xbp1^+/−^*) exhibited increased sensitivity to alcohol based on the amount of acinar cell damage compared to WT mice [7]). These findings suggest that events that alter the UPR may predispose individuals to ethanol-initiated pancreatitis. In addition, all three pathways of the UPR are activated in response to experimentally-induced pancreatitis [11], [12], [13].

MIST1 (also known as bHLHA15) is a basic helix-loop-helix (bHLH) transcription factor required for complete acinar cell maturation and a target for XBP1 transcriptional regulation [14], [15]. Work from our laboratory has shown that MIST1 is crucial to the maturation and function of secretory acinar cells [16], [17]. Ablation of the *Mist1* gene in mice (*Mist1^−/−^*) leads to altered acinar organization, exocytosis and Ca^2+^ handling [16], [17], [18], [19]. *Mist1^−/−^* mice also show increased pancreatic injury and decreased activation of the UPR in response to cerulein-induced pancreatitis (CIP) [11].

Based on these studies, we hypothesized that *Mist1^−/−^* mice would be more sensitive to chronic ethanol feeding. We report here three major findings. First, *Mist1* ***^−/−^*** mice develop periductal accumulations of inflammatory cells in response to ethanol feeding that are not observed in congenic mice. Second, wild type mice exposed to feeding of diets high in ethanol and/or fat resulted in increased levels of IRE1 and PERK signaling, indicating that the UPR is activated in pancreatic tissue by conditions that are risk factors for pancreatitis. Third, exposure to ethanol resulted in decreased UPR activation in *Mist1^−/−^* mice. Therefore, an absence of MIST1 function may be a link to increased susceptibility to pharmacological and environmental factors that promote pancreatic injury.

## Methods

### Ethics statement

All procedures were approved by the Animal Care Committee at the University of Western Ontario (Protocol # 2008-116) and mice were handled according to regulations established by the Canadian Council for Animal Care to ameliorate suffering in these animals.

### Animal handling, feeding and cerulein induced pancreatitis

Male *Mist1^−/−^* [17] and congenic C57 Bl6 mice were housed individually and fed a Lieber-DeCarli ethanol (LDC-E; diet #L10016, Research Diets, New Brunswick, NJ) diet *ad libitum* for 6 weeks that consisted of 36% of calories from ethanol [20]. This diet also contained 36% of kcal from fat. As a control, mice were fed a diet that replaced ethanol kcal with isocaloric maltodextrin (LDC-HF; diet #L10015, Research Diets), or breeding chow that had a lower composition of fat (22% kcal; Global 2019 Rodent Diet, Teklad Diets, Madison, WI). For comparison of diets, see Table 1. Animals were weighed daily or weekly and food intake measured daily.

**Table 1:** Comparison of LDC-HF and LDC-E diets to Breeder Chow.

Component	Research Diet #L10015*	Research Diet #L10016*	Teklad Extruded Rodent Diet*
Protein	17	17	23
Carbohydrate	47	11	55
Fat	36	36	22
Ethanol	0	36	0

*values represent %kcal that each component contributes to the diet.

### Serum amylase assay

Blood serum was obtained through cardiac puncture, placed on ice for 20 minutes and centrifuged at 5000 g for 15 minutes at 4°C. Serum amylase levels were determined using a *Phadebas* amylase detection kit (Pharmacia Diagnostics, Dorval, QC) as per manufacturer's instructions.

### Antibodies

Primary antibodies used included rabbit antibodies directed against amylase (dilution 1∶1000; Calbiochem, San Diego, CA), BiP/GRP78 (1∶1000; Cell Signalling Technology, Pickering, ON), Carboxypeptidase (1∶1000; Cedarlane Laboratories, Hornby, ON), CD4 (1∶500, BD Pharmingen, Mississauga, ON), total eIF2α (1∶500; Cell Signalling), GADD34 (1∶500; Santa Cruz Biotechnologies, Santa Cruz, CA). LC3-I/II (1∶500; Cedarlane), phospho (p) PERK (1∶500; Cell Signalling), peIF2α (1∶500; Cell Signalling), XBP1 (1∶500; Santa Cruz) and β-actin (1∶500; Santa Cruz), a mouse antibody raised against insulin (1∶1000; Sigma, St. Louis, MO), and a goat antibody against β-tubulin (1∶500; Santa Cruz). Secondary antibodies were conjugated to fluorescein isothiocyanate (FITC; dilution 1∶250; Sigma), tetramethyl rhodamine iso-thiocyanate (TRITC; 1∶250; Sigma) for immunofluorescent analysis or, in the case of Western blotting, horse radish peroxidase (HRP; 1∶10,000; Sigma).

### Protein isolation and Western blot analysis

Dissected pancreatic tissue was immediately frozen in liquid nitrogen. Tissue was homogenized and sonicated in isolation buffer consisting of 1 M Tris (pH 7.2), 1 M MgCl_2_, 1 M CaCl_2_, 1 M DTT, 5% (wt/vol) Nonidet P-40, 30 mM NaF, 2 mM NaVO, 5 µM Leupeptin, 5 µM Chymostatin, 5 µM Pepstatin, 1 mM PMSF and dH_2_0. All reagents were purchased from Fisher Scientific (Ottawa, ON). Approximately 500 µL of the buffer was used per 100 mg of tissue. Protein samples were quantified using a Bradford protein quantification assay (Bio-Rad Laboratories, Richmond, CA).

For Western blot analysis, 50 µg of protein was resolved by SDS-PAGE and transferred to a PVDF membrane (Bio-Rad Laboratories) via wet transfer. The membrane was incubated with blocking buffer (5% Non-fat dry milk in PBS with 0.1% Tween) for 30 minutes, followed by incubation in primary antibody diluted in blocking buffer for 1 hour, and incubation in secondary antibody diluted in blocking buffer for one hour. To detect signal, the membrane was incubated in enhanced chemiluminescent substrate (Perkin Elmer, Woodbridge, ON) and exposed to X-ray film. Alternately, the membrane was imaged using the VersaDoc 3000 imaging system (Bio-Rad Laboratories) on the chemiluminscence channel and subsequently analyzed. Densitometric analysis was performed on images obtained on the VersaDoc imaging system using ImageLab Software (Bio-Rad). Artificial bands were overlaid onto the protein bands in the image and adjusted accordingly. Band volumes were determined by the software based on relative pixel intensities.

### Immunofluorescent and Histological analysis

Following dissection, the pancreas was placed into Shandon cryomatrix solution (Fisher Scientific, Ottawa, ON) and frozen in liquid nitrogen as described in [17]. Frozen sections were sectioned to 7 µm using a Shandon cryostat (Fisher Scientific) and intracellular staining performed as previously described [21]. TUNEL analysis was performed as described (11). For histological analysis, pancreatic tissue was rinsed in PBS, fixed in 4% formaldehyde for 24 hours and processed for paraffin sectioning. Tissue was sectioned to 7 µm and stained with Gomori's trichrome.

### RNA isolation and RT PCR

Total pancreatic RNA was isolated using TRIzol (Invitrogen, Carlsbad, CA) as previously described (17). Reverse Transcriptase (RT) PCR was performed with 1 µL cDNA using primers for *β-actin* and *Xbp1* (Sigma) on a GeneAmp® PCR system 9700 (Perkin Elmer Applied Biosystems, Woodbridge, ON). Primers to amplify *Xbp1* and *β-actin* were based on sequences obtained from NCBI (http://www.ncbi.nlm.nih.gov/tools/primer-blast). Primer sequences to amplify for *Xbp1* and *Mist1* were:

*Xbp1* forward: 5′ AAACAGAGTAGCAGCGCAGACTGC 3′

*Xbp1* reverse: 5′ TCCTTCTGGGTAGACCTCTGGGAG 3′

*Mist1* forward: 5′ GTGGTGGCTAAAGCTACGTG 3′

*Mist1* reverse: 5′ GACTGGGGTCTGTCAGGTGT 3′

These primers produced amplicon sizes of 289 bp and 263 bp for *uXbp1* and *sXbp1*, respectively, and 212 bp for *Mist1*. To calculate the ratio of *sXbp1* to *uXbp1*, pixel intensity of the *sXbp1* band was divided by the pixel intensity of the *uXbp1* band, obtained using ImageLab software (Bio-Rad) following visualization in the VersaDoc 3000 imaging system.

Primer sequences to amplify for *β-actin* were:

*β-actin* forward: 5′ ATGGAGAAGATCTGGCAC 3′

*β-actin* reverse: 5′ CGTCACACTTCATGATGG 3′

These primers produced amplicon sizes of 212 bp.

### Statistical analysis

Statistical tests including a student's T-test, One-way Analysis of Variance (ANOVA) or Two-way ANOVA, followed by Bonferroni or Tukey post hoc tests were performed via GraphPad Prism 4.0 software (GraphPad Software, San Diego, CA) and used to obtain quantitative data.

## Results

The inability of ethanol to induce pancreatic injury in rodents suggests additional events exist that affect the human response to alcohol consumption. Since our previous work indicated that an absence of MIST1 increased sensitivity to experimentally induced pancreatitis, the effects of long term ethanol feeding of *Mist1^−/−^* mice were examined. C57 Bl6 and congenic *Mist1^−/−^* mice were placed on an ethanol-supplemented liquid Lieber-DeCarli (LDC) diet, which contains 36% kcal from ethanol and 36% kcal from fat. As a control, animals were either placed on an identical diet with the calories from ethanol replaced by carbohydrates (LDC-HF), or maintained on regular breeding chow (22% kcal from fat). See Table 1 for components of each diet. The rationale for including two control diets was to exclude the possibility that the high fat content of the LDC-E diet accounted for any effects observed.

**Figure 1:**
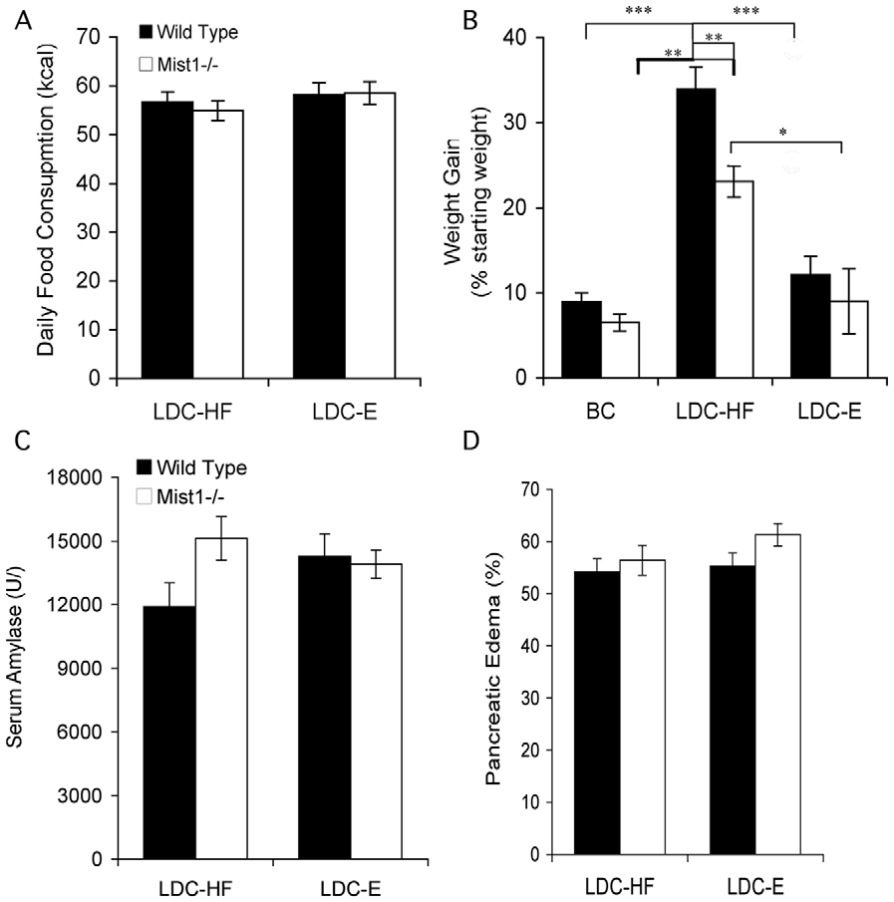
WT and *Mist1^−/−^* mice show no overt response to ethanol feeding. Caloric intake (**A**) and weight gain (**B**) was monitored over six weeks of feeding with a Lieber-DeCarli Ethanol (LDC-E), LDC high fat (LDC-HF) or breeding chow (BC) diet. Letters represent statistically significant differences between groups (see text for details). Groups were compared using Two-way ANOVA and Bonferroni post-hoc test. *P<0.05, **P<0.01, ***P<0.001. n = 7 (BC), n = 11 (LDC-HF), n = 12 (LDC-E). The LDC-HF diet resulted in increased weight gain in both genotypes. At six weeks, mice were killed and serum amylase (**C**) and pancreatic edema (**D**) calculated. No significant differences were observed between genotypes or treatments. n = 11 (LDC-HF), n = 12 (LDC-E).

WT and *Mist1^−/−^* mice on the LDC-E and LDC-HF diets consumed equivalent kcal amounts throughout the length of the study, with minimal individual variability in daily caloric intake (**Figure 1A**). Due to the solid nature of the breeder chow food, it was difficult to get an accurate measurement of caloric consumption in this group. Even with similar kcal consumption, the rate of weight gain was significantly higher in LDC-HF fed mice for both genotypes compared to LDC-E counterparts **(** **Figure 1B** **)** or breeder chow fed mice After six weeks of ethanol feeding, LDC-HF fed mice weighed significantly more than LDC-E counterparts. Interestingly, WT mice on the LDC-HF diet gained significantly more weight than *Mist1^−/−^* mice. Analysis of serum amylase levels (**Figure 1C**) and pancreatic edema (**Figure 1D**), which are markers of pancreatic injury, showed no significant differences for either genotype. These results indicate that only LDC-HF fed mice gained significant weight throughout the course of this study.

**Figure 2:**
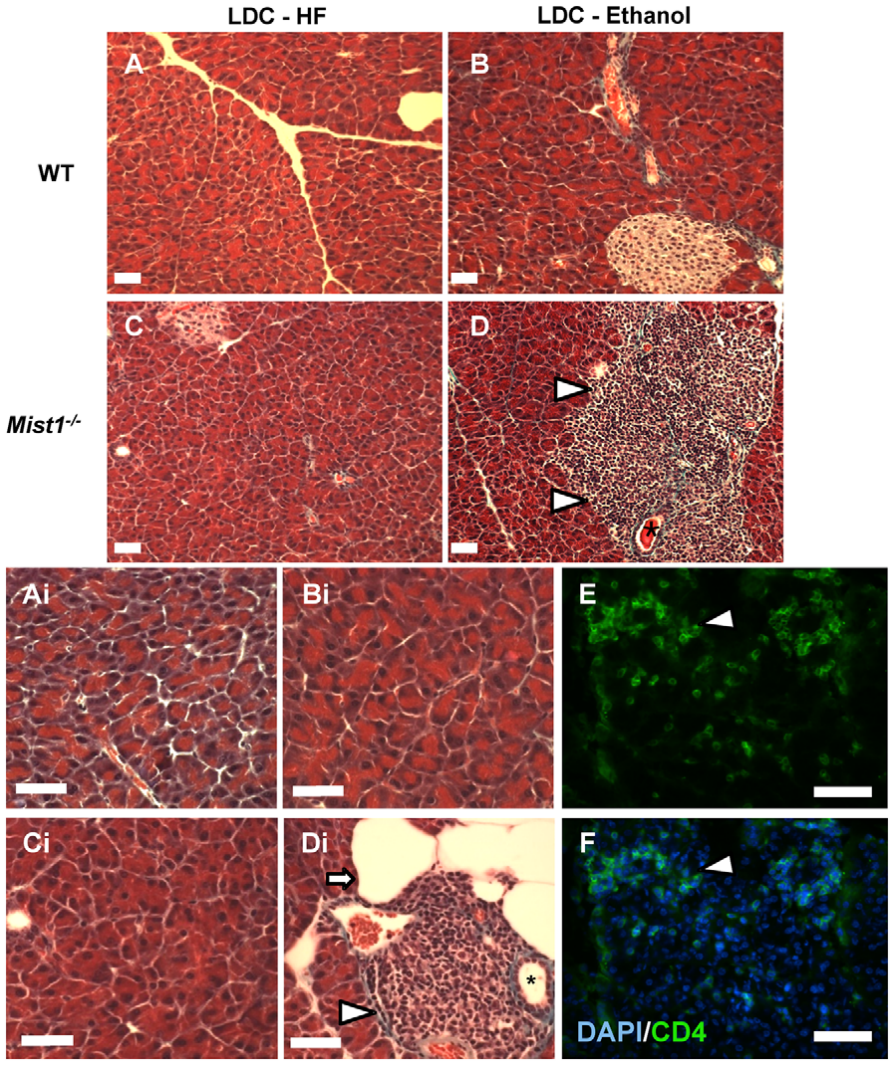
*Mist1^−/−^* pancreata develop periductal accumulations of inflammatory cells in response to ethanol feeding. Representative photomicrographs of Gomori's trichrome stained pancreatic sections from WT (**A, B**) and *Mist1^−/−^* mice (**C, D**) fed a LDC-HF (**A, C**) or LDC-E (**B, D**) diet for 6 weeks. Higher magnification images from the same sections (**Ai–Di**) were used to highlight specific morphological events. Cellular accumulations (arrowhead) surrounding ducts (*) and adipose accumulations (arrow) were observed only in LDC-E fed *Mist1^−/−^* mice. (**E, F**) Immunofluorescent analysis for the T-lymphocyte marker CD4 (**E**) indicated that these accumulations consisted of lymphocytes (arrowhead). Sections were co-stained with DAPI (**F**) to reveal all cells. Magnification bars = 40 µm.

Gomori's trichrome stained pancreatic sections revealed no signs of pathological damage or cell disorganization in LDC-E or LDC-HF fed WT mice (**Figure 2A, B**). LDC-HF fed *Mist1* ***^−/−^*** mice showed acinar disorganization similar to that reported as a result of targeted ablation of MIST1 (**Figure 2C**; [17]). Importantly, 75% of ethanol fed *Mist1* ***^−/−^*** mice (9/12) showed obvious periductal infiltration of inflammatory cells (**Figure 2D**) while 0/23 of the WT mice and 0/11 LDC-HF fed *Mist1* ***^−/−^*** mice showed any signs of inflammation. IF analysis with the T lymphocyte marker, CD4, confirmed these accumulations to be lymphocytes (**Figure 2E, F**) and also revealed smaller accumulations of CD4 positive cells in most LDC-E fed *Mist1^−/−^* pancreatic tissue that were not evident in H&E stained tissue (**Figure 2E, F**). Similar staining of WT (0/23) and LDC-HF *Mist1^−/−^* (0/11) groups showed no such CD4+ cells within the pancreatic interstitium (data not shown). While trichrome histological staining did not reveal significant pancreatic fibrosis, 50% of ethanol fed *Mist1^−/−^* mice (6/12) also showed accumulation of adipocytes (compared to 0/23 of the WT mice and 0/11 LDC-HF fed *Mist1* ***^−/−^*** mice), often located adjacent to the site of inflammatory infiltrate (**Figure 2D** **i**) that were not observed in any other group. Therefore, a relatively short exposure to ethanol promoted localized inflammation only in *Mist1^−/−^* pancreatic tissue.

Recent reports have shown that chronic ethanol exposure leads to increased accumulation of sXBP1 in mouse pancreatic acinar cells [7] and ethanol can potentiate ER-stress induced cell death in cultured neurons and induce oxidant stress in rat pancreatic acinar cells as a consequence of its metabolism [22]. *Xbp1^+/−^* mice show increased inflammation and pancreatic injury upon ethanol exposure, suggesting the UPR acts in a protective fashion to prevent ethanol-induced damage [7]. Therefore, we examined the UPR in response to ethanol feeding.

**Figure 3:**
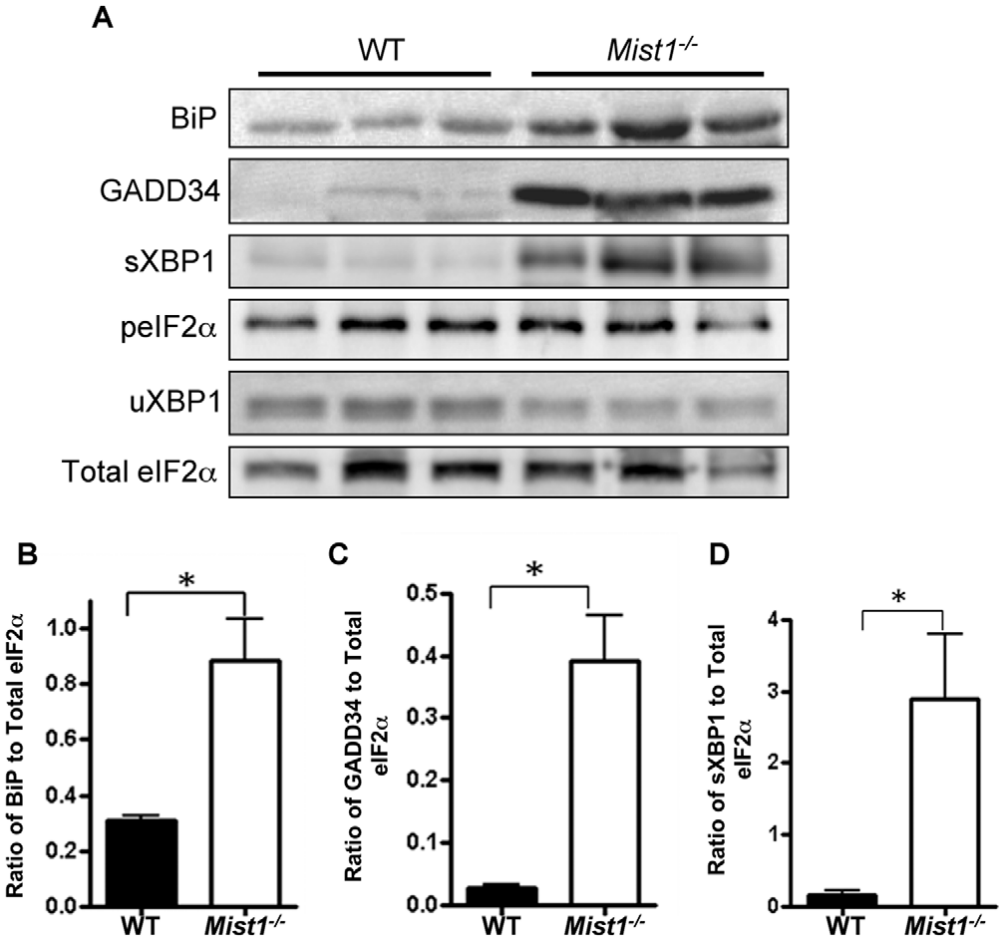
*Mist1^−/−^* pancreatic tissue exhibits increased activation of the UPR. (**A**) Western blot analysis of key UPR markers in 2 month old WT and *Mist1^−/−^* whole pancreatic lysates. *Mist1^−/−^* extracts show significantly increased accumulations of BiP/GRP78 (**B**), GADD34 (**C**) and sXBP1 (**D**), but not peIF2α (p = 0.743) or uXBP1 (p = 0.532) relative to WT pancreatic tissue. *P<0.05. n = 3.

Initially, UPR activity was compared between two month-old WT and *Mist1^−/−^* pancreatic tissue, which corresponded to a time just prior to initiating ethanol exposure. To assess activation of the UPR, the accumulation of BiP/GRP78, GADD34 (growth arrest and DNA damage inducible 34), XBP1 and peIF2α was examined by western blot analysis. BiP/GRP78 (**Figure 3A, B**), GADD34 (**Figure 3A, C**), and sXBP1 (**Figure 3A, D**) were all increased in pancreatic tissue of *Mist1^−/−^* mice compared to age-matched WT mice, while levels of uXBP1 and peIF2α remained unchanged (**Figure 3A**). This lack of higher peIF2α accumulation in *Mist1^−/−^* pancreatic tissue is likely due to the increased accumulation of GADD34, which promotes dephosphorylation of peIF2α and acts as negative feedback on PERK signaling [23].

**Figure 4:**
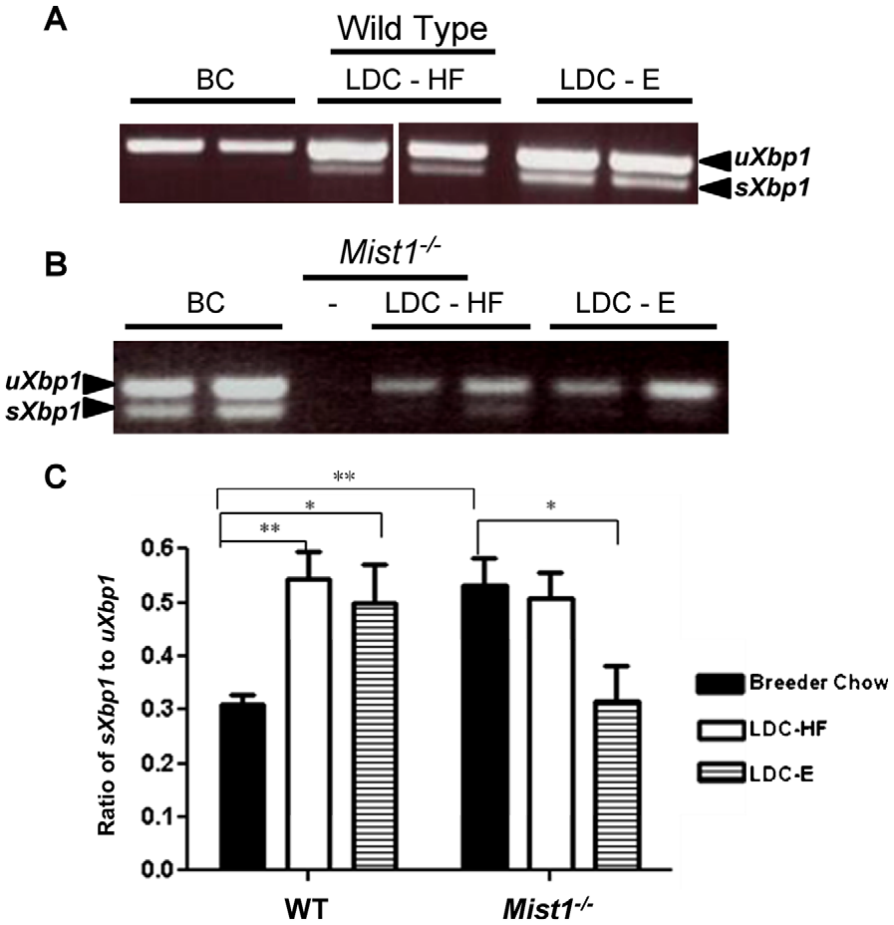
Ethanol feeding leads to increased accumulation of spliced (s) *Xbp1* only in WT pancreatic tissue. RT-PCR analysis of *Xbp1* splicing in WT (**A**) and *Mist1^−/−^* (**B**) mice fed breeder chow (BC), LDC-E or LDC-HF diets revealed accumulation of spliced (s) *Xbp1* mRNA in response to both LDC-E and LDC-HF diets in WT pancreatic tissue. *Mist1^−/−^* mRNA samples showed *sXbp1* accumulating in BC fed mice and decreased *Xbp1* accumulation in LDC-HF and LDC-E mice. (**C**) Quantification of the ratio of spliced (*s*) *Xbp1* to unspliced (*u*) *Xbp1* as determined by densitometric analysis of images in A. *Mist1^−/−^* mice showed increased amounts of *sXbp1* relative to WT mice when fed the BC diet. LDC-HF and LDC-E diets led to a significant increase in *Xbp1* splicing while pancreatic tissue from LDC-E fed *Mist1^−/−^* mice showed decreased amounts of splicing. Groups were compared using One-way ANOVA and a Tukey post-hoc test: *P<0.05, **P<0.01. n = 3 (BC), n = 6 (LDC-HF & E).

**Figure 5:**
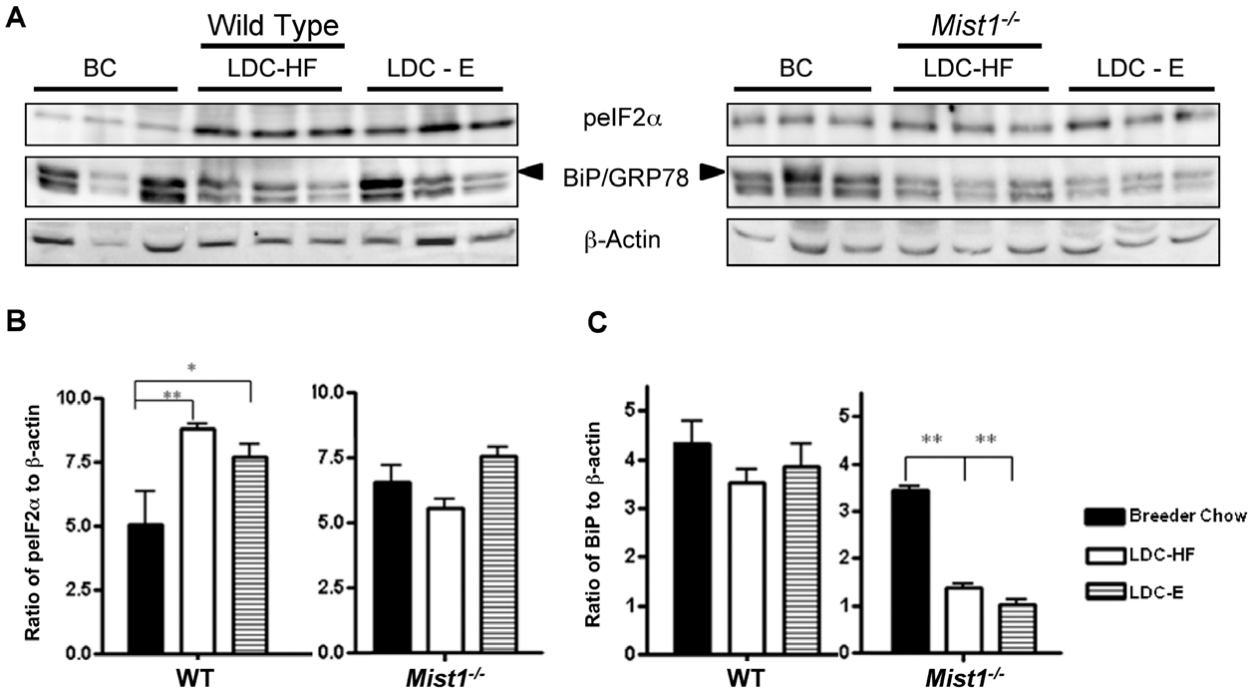
High fat and ethanol diets activate PERK signaling in pancreatic acinar cells. (**A**) Western blotting analysis for peIF2α, BiP/GRP78 (upper band) or β-actin (as a control) in pancreatic tissue of WT or *Mist1^−/−^* mice fed breeder's chow (BC) or LDC-E or LDC-HF diets for 6 weeks. Quantitative analysis of peIF2α (**B**) or BiP/GRP78 (**C**) accumulation from (A) relative to β-actin accumulation. LDC-E and LDC-HF diets lead to significant increases in peIF2α compared to BC diets in WT mice but not in *Mist1^−/−^* tissue. Conversely, while BiP/GRP78 levels are unchanged in WT mice, they decrease upon exposure to LDC diets in *Mist1^−/−^* mice. Groups were compared using One way ANOVA and a Tukey post-hoc tests: *P<0.05, **P<0.01. n = 3 (BC); n = 6 (LDC-HF & E).

**Figure 6:**
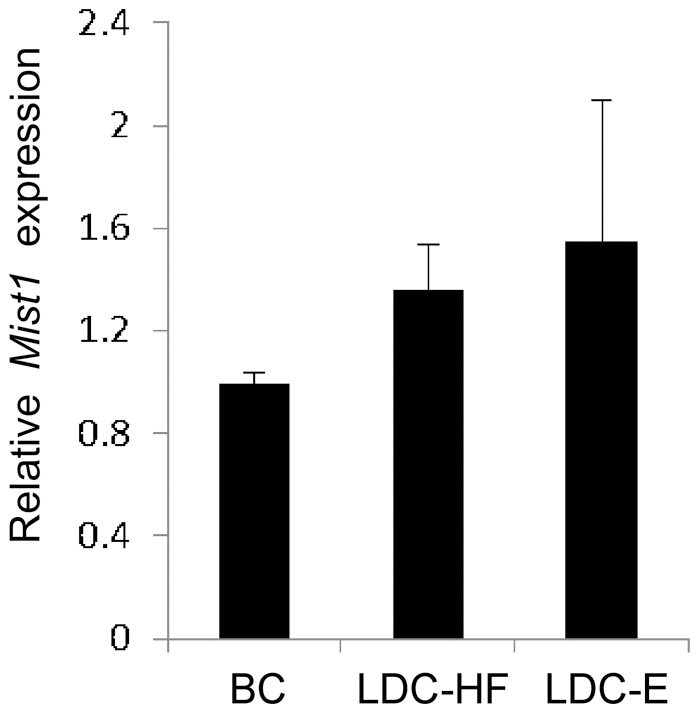
Mist1 expression is not altered in response to ethanol feeding. Quantitative RT-PCR shows no difference in *Mist1* levels in response to either LDC-E or LDC-HF diets. Error bars = mean ± SE (n = 3).

We next assessed UPR activity following exposure to ethanol and high fat diets. As previously documented, splicing of *Xbp1* was significantly enhanced in response to LDC-E feeding in WT mice [7]. Surprisingly, there was an increase in the ratio of *sXbp1/uXbp1* in LDC-HF fed mice as well indicating that a diet high in fat also activated the IRE1 pathway of the UPR (**Figure 4A, C**). A similar observation was observed for the PERK signaling pathway as increased accumulation of peIF2α was observed in LDC-HF and LDC-E diets (**Figure 5A, B**). No change was observed for BiP/GRP78 accumulation (**Figure 5A, C**). Conversely, examination of *Xbp1* splicing in *Mist1^−/−^* pancreas revealed a decrease of *Xbp1* splicing only in response to ethanol feeding, suggesting decreased IRE1 signaling in this group (**Figure 4B, C**). Interestingly, both LDC-HF and LDC-E diets reduced overall *Xbp1* accumulation, which is a target of ATF6α trans-activation [24]. Another target of ATF6α, BiP/GRP78 [24] also decreased in LDC-E and LDC-HF fed *Mist1^−/−^* mice (**Figure 5A, C**), supporting the findings that *Mist1^−/−^* mice had a reduction in ATF6 signaling as a result of ethanol or high-fat consumption. Finally, levels of peIF2α did not increase in LDC-HF or LDC-E fed *Mist1^−/−^* mice (**Figure 5A, B**) as observed for WT counterparts suggesting that PERK signaling was either at maximal activity already, or could not be activated further, possibly due to the increased levels of GADD34. These findings suggested that activation of the UPR was a consequence of a number of adverse dietary conditions, and indicated that all three pathways of the UPR were altered in *Mist1^−/−^* acinar cells upon exposure to diets that predispose individuals to pancreatitis. Previous work indicated that XBP1 targets the *Mist1* gene to activate transcription. Therefore, it is possible that MIST1 helps mediate the protective effect of XBP1 during ethanol exposure. However, while qRT-PCR analysis showed a trend towards increased *Mist1* mRNA accumulation in response to LDC-HF or LDC-E diets, these differences were not significant (**Figure 6**). Therefore, the increased sensitivity to alcohol observed in *Mist1^−/−^* pancreata does not appear to be due to the loss of XBP1 activating *Mist1* to increase granule compartmentalization.

**Figure 7:**
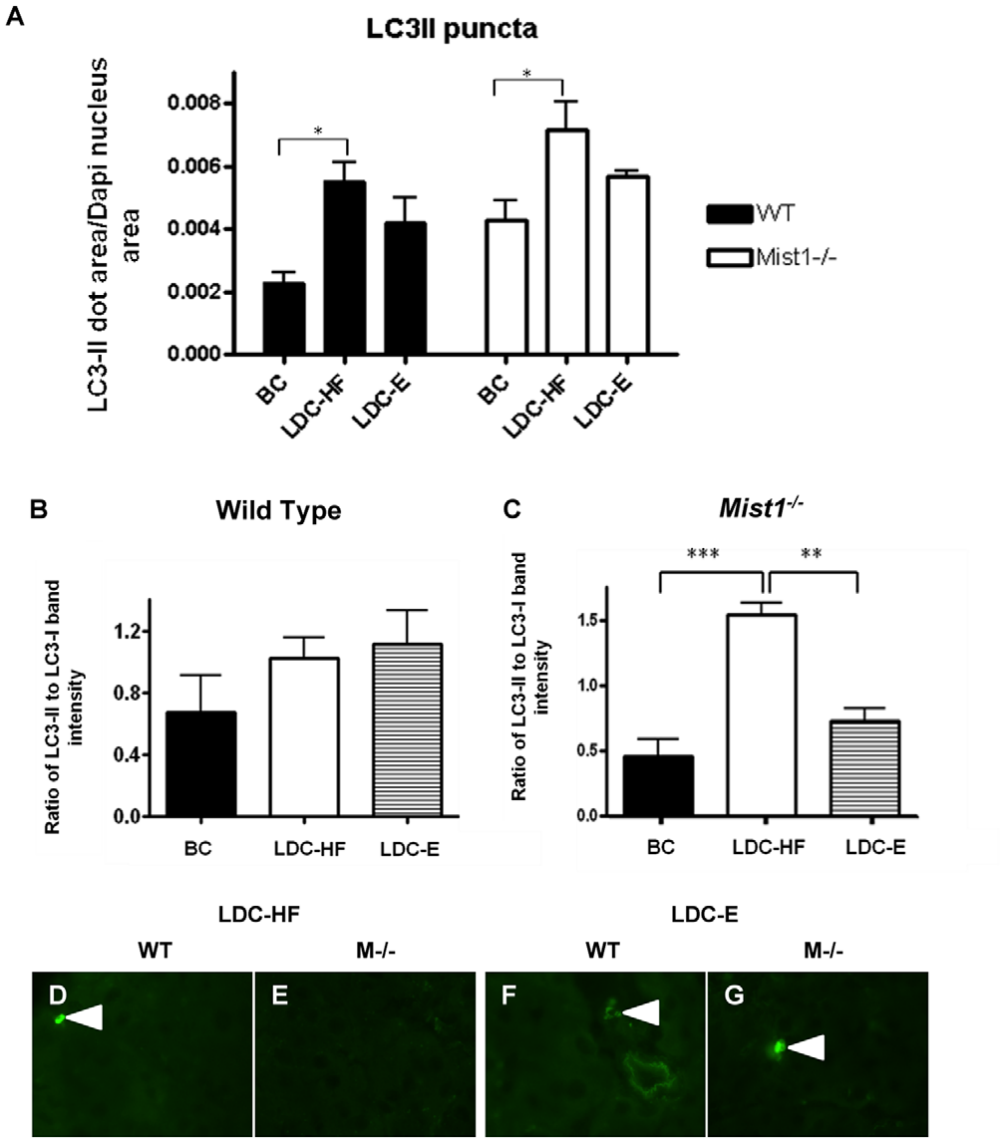
Ethanol-fed *Mist1^−/−^* mice show limited increases in autophagy and apoptosis. (**A**) Quantitative analysis of LC3-II puncta, shown as the area of LC3 dots per area of DAPI stained nuclei. Groups were compared using One-way ANOVA and a Tukey post- hoc test: *P<0.05. n = 3 (BC), n = 5 (LDC-HF & LDC-E). Quantification of the LC3-II to LC3-I ratio by densitometric analysis of western blots for WT (B) and *Mist1^−/−^* mice fed breeder's chow (BC), or diets high in fat (LDC-HF) or ethanol (LDC-E). *P<0.5, n = 3. (**D–G**) Images from TUNEL analysis performed on pancreatic tissue from WT and *Mist1^−/−^* mice showed minimal (<<1%) TUNEL+ nuclei (arrows) under all dietary conditions.

Finally, as a functional readout for the UPR, acinar cell autophagy was examined through IF analysis of LC3 lipidation (**Figure 7**). LC3-I is a protein that is normally found in the cytosol, which upon induction of autophagy, is lipidated to LC3-II and associated with membranes of autophagic vacuoles, being recognized as puncta in IF [25]. Autophagy can be triggered by the UPR to allow for the re-use of micromolecular nutrients under conditions of stress, thereby enhancing cell survival [25], [26]. Consistent with increased UPR activity, WT mice fed LDC-HF diet showed a significant increase in acinar autophagy compared to breeder chow fed controls, as determined by IF analysis of area of LC3-II puncta as a percent of the acinar cell (**Figure 7A**). The LDC-E diet only led to a moderate increase in autophagy in WT mice (**Figure 7A**). In *Mist1^−/−^* mice, analysis of LC3-II puncta revealed that the LDC-HF diet caused a significant increase in autophagy, while ethanol had no significant effect (**Figure 7A** **)**. We next analyzed autophagy via an alternative method, comparing the ratio of LC3-II:LC3-I by western blot analysis (**Figure 7B, C**). WT mice showed slightly elevated, but not statistically significant different amounts of LC3-II in response to LDC-HF or LDC-E diets (**Figure 7B**). In *Mist1^−/−^* mice, the LDC-HF diet showed a significant increase in autophagy that was not apparent in LDC-E fed mice (**Figure 7C**). This suggests that while the activation of the UPR in response to LDC-E and LDC-HF diets was affected in *Mist1^−/−^* pancreatic tissue, only the inability to activate IRE1 signaling and trigger autophagy upon ethanol exposure correlates to focal inflammation observed in LDC-E fed *Mist1^−/−^* mice. To determine if the increased autophagy was protecting against cell death, we performed TUNEL analysis to determine the amount of acinar cell apoptosis occurring in response to the LDC-E and LDC-HF diets (**Figure 7D–G**). With the exception of one LDC-E fed *Mist1^−/−^* mouse, minimal apoptotic cells were observed (<<1%), suggesting that apoptosis was not occurring in any situation. It was unclear why only one mouse showed significant amounts of apoptosis (>25% of cells were TUNEL positive), but it is possible that longer time periods of ethanol treatment may further exacerbate the *Mist1^−/−^* phenotype.

## Discussion

Pancreatitis is a debilitating disease that targets the exocrine pancreas. While chronic alcohol abuse is a leading cause of pancreatitis in humans, animal models of alcoholic pancreatitis indicate that additional factors must be present to promote pancreatic injury associated with alcohol. Alcohol sensitizes the pancreas by altering Ca^2+^ and NFκB signaling, and also leads to spatial alterations in SNARE-mediated regulated exocytosis [5], [27]. More recent studies have suggested that chronic alcohol exposure leads to activation of the ER stress response and that reductions in *Xbp1* activation enhance the effects of alcohol on the pancreatic response [7]. Here, we provide evidence that absence of the exocrine specific transcription factor, MIST1, leads to increased sensitivity to ethanol feeding coupled with decreased activation of the UPR, a pathway that reduces susceptibility to ethanol damage [28]. These results suggest that mutations that affect MIST1 expression or function may increase sensitivity to adverse environmental conditions that promote pancreatitis in humans.

Previous studies have identified a number of genes that are linked to the development and susceptibility to pancreatitis. Mutations in *PRSS1* (*cationic trypsinogen* gene) are the underlying cause for hereditary pancreatitis [29] and mutations in *SPINK1 (serine protease inhibitor Kazal type I*) are often found in patients with chronic or acute forms of the disease [30]. Mutations in *CTRC* (*chymotrypsinogen C)* and *CFTR (cystic fibrosis transmembrane receptor)* have also been associated with increased risk for pancreatitis [31], [32]. However, there is still a significant contribution to the prevalence of pancreatitis from unknown genetic factors. The results from this study suggest that mutations in MIST1 could also be linked to the disease. A complete absence of MIST1 is not deleterious to overall health, reproductive ability, viability, and survival of the mice [17], [33] indicating that altered MIST1 function can go unnoticed for long periods of time. While a small percentage of *Mist1^−/−^* mice do develop more overt fibrosis and inflammation characteristic of chronic pancreatitis, the majority (95%) of the mice are relatively unaffected.

These studies also highlight the importance of *Mist1^−/−^* mice as a useful tool for studying factors that promote or protect against pancreatic injury. Our previous work has shown a marked increase in pancreatic damage associated with experimentally-induced pancreatic injury [11]. This study indicates that relatively short exposure (6 weeks) of *Mist1^−/−^* mice to ethanol can promote increased inflammation. While other models of ethanol exposure have been used, including intravenous infusion of ethanol into the circulation, the LDC-E diet mimics the level of circulating ethanol levels found in alcoholics [5]. The time frame we have used, 6 weeks, is enough to sensitize WT pancreatic tissue to CIP, but does not lead to overt effects on pancreatic function or morphology. While the absence of MIST1 results in periductal accumulations of inflammatory cells and increased adipose depositions within the tissue, there is not an overt response such as elevated levels of circulating amylase or extensive fibrosis associated with pancreatitis. However, it is likely that the limited duration of exposure to the *Mist1^−/−^* pancreas or the age of the animals within this study - 2 to 4 months of age at the beginning of treatment – contribute to this mild phenotype. Likely, longer treatment times or using older *Mist1^−/−^* mice would show a more dramatic response. Indeed, aged *Mist1^−/−^* mice (18 months of age) show similar pancreatic periductal accumulations as observed in the LDC-E treated mice (data not shown).

The underlying mechanism behind the increased sensitivity of *Mist1^−/−^* to ethanol remains elusive. A compelling target for this mechanism is the UPR, a protective cellular mechanism that protects against ethanol induced pancreatic damage in mice [7]. The inability to activate this pathway in response to environmental stressors would be detrimental to the pancreas, and our previous work showed that *Mist1^−/−^* mice exhibited increased severity following CIP coincident with a greatly reduced UPR response, We have shown here that *Mist1^−/−^* pancreatic tissue has a higher levels of UPR activity compared to WT mice under normal conditions, However, unlike the WT response to LDC-E and LDC-HE diets, there is no further increase in UPR activity in response to ethanol feeding. Indeed, both *Xbp1* splicing and BiP accumulation is reduced in LDC-E fed *Mist1^−/−^* mice. Whether this suggests that *Mist1^−/−^* acinar cells are already at maximal levels of activation of the UPR prior to dietary challenges, or that a second stress causes suppression on the UPR in these mice is unclear. In either scenario, it suggests that *Mist1^−/−^* acinar cells exhibit a level of UPR activity that cannot be further amplified or maintained during a second stress event. This makes the tissue more susceptible to damage.

The finding that inflammation was observed only in the LDC-E fed mice suggests that the specific loss of spliced *Xbp1* may be responsible for this outcome. While recent studies show that deletion of even a single copy of XBP1 leads to significant tissue damage, fibrosis and inflammation in response to chronic ethanol exposure, these outcomes were not observed in LDC-E treated *Mist1^−/−^* mice even though we show a decrease in both total and spliced XBP1 accumulation. However, the differences may be attributed to the model of ethanol exposure used in each study. Lugea et al (2011) provided ethanol through continuous intragastric infusion, which likely increased the overall exposure to ethanol within these animals. Given that the *Mist1* gene is a target for XBP1 transcriptional regulation [14], [15], the simplest model would suggest that MIST1 mediates part of XBP1's protective effect against ethanol feeding. However, we observed no statistically significant increase in *Mist1* expression in the presence of increased XBP1.

An alternative scenario is that *Mist1^−/−^* acinar cells adapt to chronic stress by reducing UPR activity relative to their basal hyperstimulated state. Previous work on the *Mist1^−/−^* phenotype has revealed intracellular activation of digestive enzymes, cell disorganization, increased autophagy and stellate cell activation, all of which will put undue stress on the tissue. We have shown elevated expression or activity of each UPR pathway with GADD34 (PERK), spliced XBP1 (IRE1) and Bip/GRP78 (ATF6) in *Mist1^−/−^* tissue. Ethanol exposure decreased the expression of BiP/GRP78 and spliced *Xbp1* over time, corresponding to appearance of inflammatory cell accumulations. We suggest that *Mist1^−/−^* acinar cells adapt to limit the deleterious consequences of chronic activation of the UPR. When a second stress, such as ethanol, is introduced, the acinar cells are unable to further activate the pathway required for protection. The elevated levels of GADD34 found in *Mist1^−/−^* tissue support such a model. GADD34 is a cofactor for protein phosphatase 1, promoting the dephosphorylation of eIF2α [23]. Decreased accumulation of *sXbp1* and BiP/GRP78 also support a model in which the UPR cannot be properly activated. Further work is required to distinguish between the two models of relationship between MIST1 and XBP1/UPR signaling.

Another key finding in this study is that a diet high in fat can also activate UPR signaling. The LDC-HF diet contains 36% kcal from fat, which is only moderately less than “western” diets used for studying obesity [34]. In LDC-HF fed mice, both PERK and IRE1 signaling were enhanced based on increased accumulation of peIF2α and increased splicing of *Xbp1*, respectively. The differences in how *Mist1^−/−^* acini respond to the LDC-E and LDC-HF diets also provide insight into the pathways that protect against injury. BiP/GRP78 and total XBP1 were reduced under conditions of ethanol or high fat feeding, but *Xbp1* splicing was reduced only in LDC-E fed *Mist1^−/−^* mice, suggesting that IRE1 signaling through XBP1 protects against ethanol's effects. XBP1 is critical for activating autophagy under ER stress conditions [35]. Autophagy induced by the UPR is crucial for cell survival and may act as either a degradation mechanism for unfolded proteins or maintain energy homeostasis to protect against cell death [35]. Hence, the inability for *Mist1^−/−^* mice to activate autophagy upon ethanol exposure may account for the differential response of these mice to LDC-E and LDC-HF diets. While one would predict that the inability to increase autophagy may lead to increased apoptosis, we did not observe such a response. However, our previous work showed that the amount of acinar cell apoptosis is less in *Mist1^−/−^* pancreata relative to WT tissue during injury (11). In that study, a decrease in apoptosis was accompanied by an increase in necrosis. If a similar response of *Mist1^−/−^* acinar cells occurred during exposure to ethanol, this would account for the negative TUNEL results and inflammatory response observed in *Mist1^−/−^* LDC-E fed mice.

The work presented here indicates that an absence of MIST1 promotes increased sensitivity to ethanol and may be an underlying genetic contributor to pancreatitis. One mechanism that may account for this sensitivity would involve MIST1's involvement, either directly or indirectly, with the UPR. *Mist1^−/−^* tissue shows an inability to further activate UPR signaling pathways in response to injurious cues. Further work to delineate the relationship between MIST1, the UPR and pancreatitis is necessary.
